# Prevalence, pattern and determinants of substance abuse among youths in a rural community of Osun State, Southwest Nigeria

**DOI:** 10.4314/ahs.v23i4.59

**Published:** 2023-12

**Authors:** Ajibola Idowu, Ayodele Olatayo Aremu, Ibukun Mary Akanbi, Gloria Eseigbe, Victoria Adewale, Loliya Awubite, Olumide Adebayo, Daniella Arisa, Blessing Adetona, Ayomikun Olaniyan, Ebunoluwa Olafisoye, Oluwaseyi Olorunshola, Jesulayomi Eyitayo, Omotolani Ogunlana, Oluwaseye Aboloye, Anatasia Mayor, Emmanuel Olatunde

**Affiliations:** Department of Community Medicine, College of Health Sciences, Bowen University, Iwo Nigeria

**Keywords:** Substance abuse, adolescents, youths, prevalence, patterns

## Abstract

**Study objectives:**

This study assessed the prevalence, patterns and factors associated with substance abuse among youths of Ejigbo community, Osun State, Nigeria.

**Method:**

This was a descriptive cross–sectional study which employed cluster sampling method to select 420 consenting youths (aged 15-24years). Data were collected using interviewer–administered, semi structured questionnaire. Descriptive and inferential statistics were carried out at p < 0.05.

**Results:**

The mean age (±SD) of the respondents was 19 ± 4.18 years. Majority (89%) of the respondents possessed good knowledge of substance abuse while 4% of them had a positive attitude towards it. Above a quarter (29.8%) of respondents had ever consumed alcoholic beverages while 12.3% of them had engaged in substance abuse. Besides alcohol, Shisha and tramadol were the most commonly abused substances in the study setting. Respondents' age (AOR=3.11;95%CI=1.67-5.24), gender (AOR=1.87;95%CI=1.53-9.25), attitude to substance use (AOR=5.90;95%3.45-10.23) and marital status (AOR=3.27;95%-CI=2.71-7.24) were the main determinants of substance abuse in the study setting.

**Conclusion:**

Respondents in the current study had good knowledge, predominantly negative attitude but a relatively high burden of substance abuse. There is urgent need for policy makers to upscale fights against the menace of substance abuse among rural Nigerian youths.

## Background

The problem of substance abuse especially among youths and young persons is a silent epidemic and a major public health problem globally. A total of 270 million people (about 5.5% of the world's population) are estimated to be using psychoactive substances globally in 2021 1. Also, not less than 35 million people are currently being affected by drug use disorders while 0.5million deaths are attributed to abnormal drug use all over the world 1.

The burden of substance abuse is humongous as about 1.3% of the global disease burden is attributed to it in 2017. It is also estimated that there are about 11 million people who inject drugs worldwide, of whom 1.4 million live with HIV and 5.6 million - with hepatitis C 1. The burden of substance abuse is also huge in sub-Saharan Africa (SSA). A systematic review by Olawole-Isaac et al.^2^, revealed the prevalence of substance abuse among adolescents in Africa to be as high as 41.6%. Alcohol is the most commonly used drug, and approximately 22.5 million adolescents (aged 12–19 years) are current drinkers in SSA 2,3

In Nigeria, drug use is a menace especially among youths. According to United Nations Office on Drugs and Crime (UNODC), there were 14.3 million drug users in Nigeria in 2018 while 3 million Nigerians suffer from drug use disorders 4. This is almost times three the international prevalence of substance use. The report also shows that there are at least 11million Cannabis users in Nigeria. A scoping review by Jatau et al., revealed a prevalence of drug abuse to be as high as 20-40% and 20.9% among Nigerian youths and students respectively 5. The UNODC also revealed that 1 in 7 Nigerians aged 15-64years are drug abusers, 1 in 4 drug abusers are women and 1 in 5 drug users suffer from the consequences of the drugs 4. Data from the National Drug Law Enforcement Agency (NDLEA) shows that all categories of illicit drugs are widely abused in all the states of Nigeria including the Federal Capital Territory (FCT) 6.

Health consequences of substance abuse are enormous ranging from bipolar affective disorders, increased tendencies for criminality, depression, hallucinations, aggression to poor academic performances. Furthermore, intravenous drug users (IDUs) constitute one of the key populations which are responsible for most cases of new HIV infections in Nigeria.

A worrisome trend is a fact that some Nigerians are involved in trafficking these illicit drugs across borders, thereby denting the image of the country at the international arena. This business thrives to inspite of the efforts of the law enforcement agents to curtail it. The annual report by the NDLEA revealed that 9444 suspected drug offenders made up of 8,535 males and 909 females were apprehended in 2019 alone 6.

Thus, we embark on this research to assess the prevalence, pattern and factors associated with substance use among rural youths in Nigeria, because of its socio-economic impacts. Most of the past Nigerian studies on substance abuse were conducted among adolescents and youths in secondary schools and higher institutions 7,8 Only a few studies have been community-based and included out-of-school youths especially in rural areas. Some studies have also indicated rural-urban variations in the pattern of substance abuse among Nigerian adolescents 9,10. It is not currently evident which of the commonly abused substances are more prevalent in the study setting being a rural community. Furthermore, the current study is imperative because there could be some local illicit substances which are more readily available for abuse by youths in rural areas but are not so popular in urban areas. There could also be some factors which can make rural youths to be more vulnerable to substance abuse than their urban colleagues. There is therefore a gap in knowledge and a dearth of information regarding the true burden, patterns and factors of substance abuse among youths in rural Nigerian communities as most of the tertiary institutions where many of the past studies were conducted are in urban areas. The current study is aimed at bridging this knowledge gap by providing comprehensive baseline data which can inform effective policy formulations to reduce the menace of substance abuse among Nigerian youths.

## Materials and methods

### Description of the study area

The study was carried out in Ejigbo community of Osun State, Ejigbo LGA, Osun State in the South Western part of Nigeria. Ejigbo LGA has a projected population of approximately 172,000 11. The majority of the population is of Yoruba ethnic group, but Hausas and Igbos also inhabit the community. The major religions are Islam, Christianity and traditional. Farming is the predominant occupation, while others engage in petty trading and artisanship. Ejigbo community has couples of guest houses with Shisha apparatus for willing users.

### Study design

This community-based study utilized cross-sectional design from August to September 2022.

### Inclusion criteria

Those included in the study were all youths aged 15-24 years who gave assent/consent and were permanent residents of Ejigbo community, Osun State.

### Exclusion criteria

Respondents within the stated age range but who had resided for a time period of less than six-months in the study setting or who were adjudged to be unable to give valid responses to our questions due to a profound illness were excluded from the study.

### Sample size calculation

Minimum sample size for the study was calculated using Leslie Kish formula for a population which is less than 10,000. A standard normal deviate at 95% confidence limit was taken as 1.96. Based on findings from a Nigerian study, we envisaged that 26.3% of our respondents may have been abusing one substance or another 7. The margin of error was set at 5%. A 10% non-response rate was assumed and appropriate corrections made. Possible cluster effect was also corrected by multiplying the estimated sample size by 1.3. Hence a minimum sample size of 413 was estimated but 420 youths participated in the study.

### Sampling method

Cluster sampling technique was used to select eligible respondents from our study population. Out of the 11 electoral wards (EWs) in Ejigbo LGA; Elejigbo, Mapo and Ejemu EWs clusters were selected using the simple random sampling method (balloting). Using our stated inclusion/exclusion criteria, eligible respondents were chosen from selected households within the chosen clusters.

### Data collection method and instrument

Data were collected using an interviewer-administered, semi-structured questionnaire adapted from the UNODC Global Assessment Programme on Drug Use Toolkit 11. The questionnaire collected information on socio-demographic characteristics of respondents, knowledge about substance abuse, attitude towards substance abuse, prevalence and patterns of substance abuse. It was translated to Yoruba language for our respondents who preferred communicating in their local language. Back translation into English language was carried out by linguistic expert, to preserve the original meanings of the questions asked. Data were collected by a group of 12 medical students on rural posting who were trained and supervised on data collection by the principal investigator.

### Pre-testing

The questionnaire was pretested among forty youths in Oko, Oyo state, (a community different from the ones used for the main study) selected using convenience sampling method. The exercise helped in assessing the appropriateness of the questions in eliciting the desired responses from our respondents. Ambiguous questions were either rephrased or removed entirely in line with our study objectives.

### Data management

Each completed questionnaire was edited daily on the field before entering into the data analysis software. All data analysis was performed using International Business Machines corporation's (IBM) Statistical Package for the Social Sciences (SPSS) version 20. Categorical data were summarized using percentages and presented using Tables, Bar and Pie Charts. Similarly, continuous data were summarized using mean and standard deviation (SD). At the bi-variate level, Chi-Squared Test was used to compare the relationships between categorical variables. A binary Logistic Regression model was built at the multivariate level. Confidence Intervals (CIs) and Adjusted Odd Ratio (AOR) were obtained to examined factors which were significantly determining substance abuse status of our respondents and the level of statistical significance was set at p<0.05.

### Definition of key variables

#### Knowledge on substance abuse

A set of eight questions were asked to assess the knowledge of respondents on risk factors, types and complications of substance abuse. Each correct point attracted two marks. Respondents' scores were summed up, rated over 16 points and converted to percentages. Respondents who scored less than 50% were classified as having poor knowledge, while those who scored 50% or higher were categorized as having good knowledge.

#### Attitude towards substance abuse

This was assessed by positively phrasing a set of 10 questions on a 5-point Likert scale which ranged from 5 (Strongly agree) to 1(Strongly disagree). The maximum score obtainable by each respondent was 50 point. The respondents' scores were rated over 50 and converted to percentages. Those who scored at least 50% of the total scores were categorized as having a negative attitude towards substance abuse, while those who scored less were classified as having positive attitude.

### Ethical consideration

Approval to carry out this study was obtained from the Research and Ethics Committee of Bowen University Teaching Hospital, Ogbomosho (NHREC/12/04/2012: BUTH/REC-692). Also, permission to conduct the study was sought from Ejigbo LGA and from the community monarch. Both verbal and written consents were obtained from study participants and parents or guardians of minors after the study objectives had been clearly explained to them. Participation in the study was entirely voluntary. Confidentiality was ensured by making the questionnaire anonymous (using codes rather than personal identifiers) and by storing the generated data in computers which were only accessible to investigators. Participants identified as substance abusers were referred to Baptist Hospital in Ejigbo for comprehensive medical/psychological attention.

## Results

Out of 420 questionnaires shared, 413 were returned satisfactorily completed (98.3% response rate). As displayed in Table 1, the mean age ± SD of the respondents was 19.01 ± 4.18. Most (60.3%) of the respondents were adolescents, 55.2% were males, 59.3% attained secondary education and 59.3% were Moslems. Most (62.2%) of the respondents live with both parents. Almost half (48%) and 52.1 % of the fathers and mothers attained secondary education respectively. Most (66.3%) of the respondents stated that they had a good relationship with their parents, and 47.2% were highly involved in religious activities.

**Table 1:** Socio-demographic characteristics of respondents

Variable	Frequency N=413	Percent (%)
**Age (Years)**		
15-19	249	60.3
20-24	164	39.7
**Mean age**	19.01	
**Sex**		
Male	228	55.2
Female	185	44.8
**Religion**		
Christianity	155	37.5
Islam	245	59.3
Traditional	13	3.2
**Marital status**		
Single	370	89.6
Married	43	10.4
**Highest level of education**		
No formal education	11	2.7
Primary	113	27.4
Secondary	245	59.3
Tertiary	44	10.6
**Living status**		
Father only	21	5.1
Mother only	58	14.0
Both parents	257	62.2
Living alone	25	6.1
Guardian/sibling	26	6.3
Friend	26	6.3
**Mother' highest education**		
No formal education	64	15.5
Primary	71	17.2
Secondary	215	52.1
Tertiary	63	15.2
**Father's highest education**		
No formal education	67	16.2
Primary	52	12.6
Secondary	198	48.0
Tertiary	96	23.2
**Relationship with parents**		
Very good	274	66.3
Good	116	28.1
Poor	23	5.6
**Involvements in religious activities**		
Very active	195	47.2
Active	182	44.1
Inactive	27	6.5
Not involved	9	2.2

In Table 2, 77.5% of the respondents were aware that some youths in the community were substance abusers. Most (78.1%) of the respondents' sources of information on substance abuse were teachers.

**Table 2:** Knowledge and attitude of respondents to substance abuse

Variable	Frequency N=413	Percent (%)
**Aware of substance abuse by youths in the community.**		
Yes	320	77.5
No	93	22.5
**Knows about dangers of substance abuse**		
Yes	358	86.7
No	36	8.7
Don't know	19	4.6
**Perceived dangers of substance abuse** *		
Mental problem	324	83.5
Violent behaviour	293	75.5
Death	279	71.9
Lack of concentration in class	262	67.5
School drop-out	274	70.6
Sexual violence	241	62.1
Other criminal tendencies	235	60.6
Others	49	12.6
**Sources of knowledge on substance abuse** *		
Teacher	313	78.1
Parents	185	46.1
Pastor/Imam	102	25.4
Friends	74	18.5
Social media	76	19.0
Others	35	8.7
**Drugs perceived to be commonly abused by youths in the community** *		
Alcohol	357	86.4
Cigarette	337	81.6
Shisha	259	62.7
Snuff	193	46.7
Cocaine	166	40.2
Tramadol	276	66.8
Marijuana/weed	295	71.4
Cough syrup/codeine	230	55.7
Cannabis	179	43.3
Prescription drugs	123	29.8
Inhalants	162	39.2
Others	220	53.3
**Overall, knowledge on substance abuse**		
Good	368	89.0
Poor	45	11.0
**Attitude to substance abuse**		
Positive	17	4.0
Negative	396	96.0

* Multiple responses allowed

**Figure 1:**
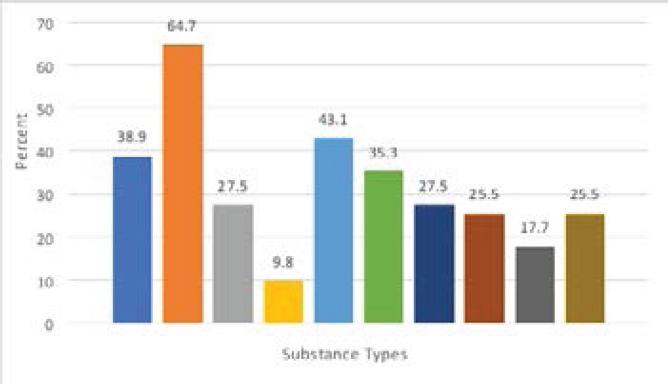
Pattern of substance abuse among the respondents

In Table 3, 29.8% of respondents had ever consumed alcohol containing drinks, palm wine was the most common alcoholic drink ever consumed, reported by 75.2% of those who had ever taken alcohol. The majority (77.2%) of those who had taken alcoholic drinks started when they were 10-19 years of age. Most (60.9%) of those who had ever consumed alcohol confirmed that the last time an alcoholic drink was taken was more than a month prior to this survey. However, 12.3% of respondents have engaged in substance abuse, with the commonest reason being ‘just to have fun’, reported by 43.1% of the substance abusers. The majority (82.4%) of the substance abusers were introduced to the substances by their friends. Shisha and tramadol were the most commonly abused substances in the study setting (Figure 1).

**Table 3:** Respondents' substance abuse status

Variables	Frequency N=413	Percent
**Ever drank alcoholic drinks**		
Yes	123	29.8
No	290	70.2
Types of alcoholic drinks ever taken *	**N = 123**	
Beer	33	25.6
Palm wine	97	75.2
Local gin (ogogoro)	21	16.3
Others	7	5.4
**Age at first shot at alcoholic drinks (Years)**	**N = 123**	
<10	11	9.0
10-19	95	77.2
20-24	17	13.8
**Last time alcoholic drink was taken**	**N = 123**	
The day of the interview	4	3.3
Past one week	21	17.1
Past one month	23	18.7
More than a month	75	60.9
**Ever taken unprescribed drugs**	**N = 413**	
Yes	51	12.3
No	362	87.7
Reasons for substance abuse*		
To be more active	18	30.5
To be more intelligent	11	18.6
Just to have fun	22	37.3
To feel happier and cool	14	23.7
Because my friends are taking it	16	27.1
To appear tough	1	1.7
Influence of parents/siblings	6	10.2
To enhance sexual performance	5	8.5
To cope with stress	8	13.6
To increase energy to perform physical tasks	6	10.2
**Have friends who are substance abusers**	**N = 413**	
Yes	101	24.5
No	312	75.5
**Person who introduced the respondent to drugs**	**N = 51**	
Friend	42	82.4
Parents	7	13.7
Siblings/relatives	2	3.9

As shown in Table 4, the proportion of substance abusers was significantly higher among males compared to females (18.4% vs. 4.9%; p = < 0.001); and among those in the 20 – 24 age group compared to those in the 15 – 19 age group (18.9% vs. 8.0%; p = 0.001). The table also shows that the proportion of substance abusers was significantly higher among those who had positive attitude towards substance abuse compared to those who had negative attitude (42.9% vs. 11.3%; p = < 0.001). Furthermore, all (100%) of the substance abusers were single rather than married and this was found to be statistically significant (p = 0.031).

**Table 4:** Factors associated with substance abuse among the respondents

Variable	Practice of Substance Abuse		Total	X^2^	P – value
	YES N= 51 (%)	NO N= 362 (%)			
**Age group**				10.794	0.001*
15 – 19	20(8.0)	229(92.0)	249		
20 – 24	31(18.9)	133(81.1)	164		
**Gender**				17.340	< 0.001*
Male	42(18.4)	186(81.6)	228		
Female	9(4.9)	176(95.1)	185		
**Religion**				0.469	0.791
Christianity	18(11.6)	137(88.4)	155		
Islam	32(13.1)	213(86.9)	245		
Traditional	1(7.7)	12(92.3)	13		
**Living Situation**				6.154	0.292
Father only	3(14.3)	18(85.7)	21		
Mother only	5(8.6)	53(91.4)	58		
Both parents	34(13.2)	223(86.8)	257		
Siblings / Guardian	4(15.4)	22(84.6)	26		
Living alone	5(20.0)	20(80.0)	25		
Living with friends	0 (0)	26(100)	26		
**Highest level of Education**				4.825	0.185
No formal education	0 (0)	11 (100)	11		
Primary	9 (8.0)	104 (92.0)	113		
Secondary	36 (14.7)	209 (85.3)	245		
Tertiary	6 (13.6)	38 (86.4)	44		
**Father's Highest level of Education**				0.803	0.849
No formal education	8 (11.9)	59 (88.1)	67		
Primary	8 (15.4)	44 (84.6)	52		
Secondary	25 (12.6)	173 (87.4)	198		
Tertiary	10 (10.4)	86 (89.6)	96		
**Mother's Highest level of Education**				2.277	0.517
No formal education	9 (14.1)	55 (85.9)	64		
Primary	5 (7.0)	66 (93)	71		
Secondary	28 (13.0)	187 (87.0)	215		
Tertiary	9 (14.3)	54 (85.7)	63		
**Ever taught about substance abuse in School**				3.336	0.067762
Yes	41 (11.3)	323 (88.7)	364		
No	10 (20.4)	39 (79.6)	49		
**Attitude towards Substance Abuse**				12.461	< 0.001*
Negative	45 (11.3)	354 (88.7)	399		
Positive	6 (42.9)	8 (57.1)	14		
**Relationship with Parents**				1.542	0.462
Very good	36 (13.1)	238 (86.9)	274		
Good	14 (12.1)	102 (87.9)	116		
Poor	1 (4.3)	22 (95.7)	23		
**Religious Involvement**				3.953	0.267
Very Active	23 (11.8)	172 (88.2)	195		
Active	21 (11.5)	161 (88.5)	182		
Inactive	4 (14.8)	23 (85.2)	27		
Not Involved	3 (33.3)	6 (66.7)	9		
**Marital Status**				4.454	0.031*
Single	51 (13.8)	319 (86.2)	370		
Married	0 (0)	43 (100)	43		

* Significant at p **<0.05**

Table 5 shows that respondents in the 20-24 age category were 3 times more likely to have abused substances compared to younger respondents (AOR=3.11;95%-CI=1.67-5.24). Male respondents were 2 times more likely to be substance abusers when compared to their female counterparts (AOR=1.87;95%CI=1.53-9.25). Similarly, respondents with positive attitude were 6 times more likely to have abused substances compared to those with negative attitude (AOR=5.90;95%3.45-10.23). Respondents who were not married were 3 times more likely to practice substance abuse compared to the married ones (AOR=3.27;95%CI=2.71-7.24).

**Table 5:** Predictors of substance abuse among respondents

Variable	Practice of Substance Abuse		OR	P – value	95%CI	AOR	P-Value	95%CI
	YES N= 51 (%)	NO N= 362 (%)						
**Age group**								
15 – 19 (R)	20(8.0)	229(92.0)						
20 – 24	31(18.9)	133(81.1)	5.34	0.001*	4.91-8.12	3.11	0.023	1.67-5.24
**Gender**								
Male	42(18.4)	186(81.6)	4.12	<0.000*	2.01-7.92	1.87	0.011	1.53-9.25
Female (R)	9(4.9)	176(95.1)						
**Attitude** **towards** **substance** **abuse**								
	45 (11.3)	354 (88.7)						
	6 (42.9)	8 (57.1)	8.46	< 0.001*	6.25-9.27	5.90	0.042	3.45-10.23
Negative (R)								
Positive								
**Marital status**			4.45	0.031*	1.89-8.62	3.27	0.013	2.71-7.24
Single	51 (13.8)	319 (86.2)						
Married (R)	0 (0)	43 (100)						

## Discussion

The current study elucidated on prevalence, pattern and related factors of substance among youths in a Nigerian rural community. Overall, 89.0% of the respondents possessed good knowledge while 96.0% of them had negative attitude towards substance abuse. Our findings agree with reports from past studies 5,12–14. However, the fact that some of the respondents in the current study had positive attitude to substance abuse calls for a more concerted efforts by relevant stakeholders, to design and implement effective behavioural change communication strategies.

More than a quarter of youths in the current study had ever taken alcohol containing drinks. This is in congruence with findings from past studies 7,15 Some Nigerian studies have reported higher prevalence of alcohol use among youths 16,17. However, the fact that above a quarter of our respondents had ever consumed alcoholic drinks is of public health significance considering the numerous complications of the substance. Cultural acceptability of alcohol use in various parties/ceremonies and the ready availability of local and refined alcoholic drinks can be facilitating factors of alcohol use by the Nigerian youths. Overall, the prevalence of substance abuse among respondents in the current study was 12.3%. This is consistent with findings from other studies 18,19. However, our finding in the current study is at variance with other studies that reported a higher prevalence of substance abuse (e.g 65% by Ogunsola et al.,)^8^. The fact that majority of respondents in the current study had religious inclinations and had strong family connectedness could have been responsible for the lower rate of substance abuse in the current study as these have been reported as strong protective factors of substance abuse 8,20. The fact that majority of the respondents possessed good knowledge of substance abuse with predominantly negative attitude to its use could have also been responsible for the lower prevalence of substance abuse in the current study population. Hence, enlightenments campaigns on risk factors and dangers against substance abuse should be further strengthened among Nigerian youths. Topics on substance abuse should be adequately incorporated into the school curricular. Establishments and maintenance of “Drug Free Clubs” should be promoted in all schools. Additionally, use of role model for advertisements of alcoholic drinks especially on sport channels should be minimized using various IEC materials.

Shisha being the most prevalent substance abused in the study setting connotes an emerging public health phenomenon among Nigerian youths. There are many club houses within the study setting providing shisha to clients. Lasebikan et al., reported 7.2% as the prevalence of shisha use in Ibadan, South-West Nigeria 17. The general perception that Shisha is cool, more acceptable and of lesser health effects compared to conventional cigarette smoking could have been responsible for the increased use in the recent past. There is need to design health educational programmes to dispel these Shisha-related myths and reduce the availabilities of shisha in club houses.

Tramadol, an opioid analgesic is also one of the most abused substances in the current study. Past studies have shown gross misuse of tramadol across the states of Nigeria 21,22. As revealed by the current study, some reasons for increased use of tramadol and other psychoactive substances by youths and young adults in Nigeria include enhancement of sexual performances, relief of stress and to have enhanced energy to perform daily activities. Hence, the law enforcement agents need to work harder in reducing availability of tramadol over-the counter in the Nigerian communities and drug traffickers should be adequately sanctioned.

Male gender was a significant risk factor for substance abuse in the current study. This is in keeping with several other studies which had revealed male preponderance to substance abuse 4,19,23 Reasons for this might be to enhance sexual performance, to have courage to approach the opposite sex or other persons as well as to improve social image. Thus, male youths should be the main target of drug use reduction strategies in Nigeria as they have the potential to badly influence their female friends. Respondents in the 20 – 24 age group were more likely to abuse substances compared to younger youths. This finding is corroborated by both UNODC and Durowade et al. 24,25. At 20-24years of age, youths are expected to have left the comforts of their parent homes, to live either with spouses, independently in school hostels or alone, fending for themselves. At this stage, some are vulnerable and may be badly influenced to adopt antisocial behaviour such as substance abuse. Consequently, there is need to prioritize youths in the 20-24 age category while designing interventions to reduce the menace of substance abuse in Nigeria.

Single status was also found to be a risk factor for substance abuse in the current study. Studies have shown that marriage causes a decrease in alcohol and substance abuse 26,27. Unmarried individuals are more at liberty to adopt free lifestyles which may include substance use behaviour compared to married people who need to show that they are responsible and act as positive role models for their wards.

## Study limitations

This study might have been affected by social desirability bias because of its social sensitivity nature. However, the fact that study objectives were clearly explained and the fact that confidentiality of information given was assured prior to each interview could have minimized this bias.

## Conclusion

The prevalence of alcohol use and substance abuse was relatively high in the study population in spite of the fact that majority of the respondents possessed good knowledge and negative attitude towards it use. Shisha and tramadol were some of the substances most commonly abused by the respondents. Gender, age and marital status of respondents were the main determinants of substances abuse in the study setting. All relevant stakeholders must work synergistically to curb the menace of substance abuse among Nigerian youths because of its serious socio-economic implications.

## References

[1] (2022). World Drug Report 2021 UNODC World Drug Report 2021: pandemic effects ramp up drug risks, as youth underestimate cannabis dangers.

[2] Olawole-Isaac A, Ogundipe O, Amoo EO, Adeloye D (2018). Substance use among adolescents in sub-Saharan Africa: A systematic review and meta-analysis. SAJCH South African J Child Heal.

[3] World Health Organization (2022). Global status report on alcohol and health 2018.

[4] UNODC (2022). Drug Use in Nigeria; Executive Summary. 2019.

[5] Jatau AI, Sha'aban A, Gulma KA, Shitu Z, Khalid GM, Isa A (2021). The Burden of Drug Abuse in Nigeria: A Scoping Review of Epidemiological Studies and Drug Laws. Public Health Rev.

[6] National Drug Law Enforcement Agency (2016). Federal Republic of Nigeria. 2016 Annual Report.

[7] Idowu A, Aremu AO, Olumide A, Ogunlaja AO (2018). Substance abuse among students in selected secondary schools of an urban community of Oyo-state, South West Nigeria: Implication for policy action. Afr Health Sci.

[8] Ogunsola OO, Fatusi AO (2017). Risk and protective factors for adolescent substance use: A comparative study of secondary school students in rural and urban areas of Osun State, Nigeria. Int J Adolesc Med Health.

[9] Azuike EC (2016). Substance abuse among secondary school students in an urban and rural local government area of Anambra state, Nigeria. International journal for medical science.

[10] Ojule IN, Te-Erebe (2022). Prevalence and predictors of substance use disorder among urban and rural secondary school students of Khana, River's state, Nigeria. Eur J Public Heal Stud.

[11] United Nations Office for Drugs and Crimes (UNODC) (2023). Global Assessment Programme on Drug Abuse.

[12] City Population (2022). Population Statistics, Charts and Map.

[13] Nebhinani N, Nebhinani M Substance-related knowledge and attitude in school and college students. German Journal of Psychiatry.

[14] Mohammed MK, Ibraheem NM, Khudhair MA (2022). Journal of Population Therapeutics & Clinical Pharmacology Knowledge and attitude of substance abuse among the youths in Tikrit Iraq. J Popul Ther Clin Pharmacol.

[15] Anetor GO, Oyekan-Thomas MF (2018). Knowledge and attitude of youths to substance abuse in Alimosho Local Government area of Lagos State. Int J Biol Chem Sci.

[16] Alpheaus CID (2020). Predictors of Alcohol Use among In-School Adolescents in a Community of Ikenne Local Government Area, Ogun State, Nigeria. Texila Int J Public Heal.

[17] Ajao Y, Olajide AB (2020). Prevalence, risk factors and perceived effects of alcohol use among young people in a rural local government area in Oyo state, Nigeria. Int J Public Heal Pharm Pharmacol.

[18] Lasebikan VO, Ola BA, Lasebikan TO (2019). Shisha smoking in selected nightclubs in Nigeria. PAMJ.

[19] Gobir A, Sambo MN, Bashir SS, Olorukoba AA, Ezeh O, Bello M (2017). Prevalence and Determinants of Drug Abuse Among Youths in A Rural Community in North Western Nigeria. Trop J Heal Sci.

[20] Uwaibi NE, Omozuwa ES, Agbonrofo-Eboigbe GE (2022). Prevalence, Sociodemograhic Characteristics and Substance Abuse among Young Persons in Edo State, Nigeria. J Appl Sci Environ Manag.

[21] Nawi AM, Ismail R, Ibrahim F, Hassan MR, Manaf MRA, Amit N (2021). Risk and protective factors of drug abuse among adolescents: a systematic review. BMC Public Health.

[22] Ohale I, Okafor CC, E N, Diwe KC, Iwu AC, Oluoha UR (2017). Socio-Demographic Determinants of Psychoactive Substance Use among Students of Tertiary Institutions in Imo State, Nigeria. J Addict Res Ther.

[23] Yunusa U (2017). Determinants of Substance Abuse among Commercial Bus Drivers in Kano Metropolis, Kano State, Nigeria. Am J Nurs Sci.

[24] Ajayi AI, Somefun OD (2020). Recreational drug use among Nigerian university students: Prevalence, correlates and frequency of use. PLoS One.

[25] UNODC (2022). World Drug Report 2018. Executive summary conclusions and policy implications.

[26] Durowade KA, Elegbede OE, Pius-Imue GB, Omeiza A, Bello M, Mark-Uchendu C (2021). Substance Use: Prevalence, Pattern and Risk Factors among Undergraduate Students in a Tertiary Institution in Southwest Nigeria. J Community Med Prim Heal Care.

[27] Njoku JC, Madukwe AU, Uwaoma NC (2015). Marital Status as a Determinant of Cognitive Behavior Therapy Outcome Among Cannabis Abusing Young Adults. Eur Sci J.

[28] Olanike A (2013). Cognitive Behaviour Therapy in the Management of Conduct Disorder Among Adolescents. Mental Disorders - Theoretical and Empirical Perspectives.

